# The Homœopathic Journal of Obstetrics, Gynæcology, and Pedology for May

**Published:** 1892-06

**Authors:** 


THE HOMŒOPATHIC JOURNAL OF OBSTETRICS, GYNECOLOGY, AND PEDOLOGY, for May, is a notable number.

It is increased in size to 112 pages, and is entirely devoted to the discussion of the question of laceration of the perineum. At the December (1891) meeting of the American Obstetrical Society, Dr. George Clinton Jeffery, of Brooklyn, N. Y., read an address entitled "A Reasonable Protest Against Immediate Perineorrhaphy," in which he took strong grounds in favor of the delayed, or secondary, operation for repair. This somewhat startling paper elicited so much disapproval among those present at the meeting that Dr. Winterburn determined to give a fair opportunity for a full expression of opinion on this important subject. The result is a Symposium of thirty papers, by prominent obstetricians and gynecologists in different parts of the country. It is rather surprising to find such a variety of opinion among men of large experience. Thus, Dr. O. S. Runnels, of Indianapolis, says: "It will be a step forward when it is established as a rule of practice that the primary operation upon the lacerated perineum is as indefensible, dangerous, and useless as is the primary operation upon the lacerated cervix. I consider it malpractice in both cases." At the other extreme of opinion there are a number of prominent men, as for instance, Prof. John Nicholas Mitchell, of Philadelphia, who says: "The duty to operate on a lacerated perineum immediately after labor is imperative," or, Prof. Sheldon Leavitt, of Chicago, who says: "I insist upon the immediate operation * * * perinei thus restored are more likely to be functionally good than those built up after a lapse of three or four months," or, Prof. George R. Southwick, of Boston, who says: "My results have been better with primary perineorrhaphy than with the secondary operation. Experience is the basis of my belief in the primary operations."

But these writers do more than express opinions. They give, each in his own way, the minute details of the primary and secondary operations. This series of papers becomes thus a most valuable guide to the general practitioner. To have the most recent views of such men as Ludlam, Danforth, Ostrom, Comstock, Green, Carleton, and many others equally prominent, brought together for comparison furnishes the most vital exposition of this subject which has ever been written.

Dr. Winterburn, in closing the discussion, discourses editorially on the subject of prevention of laceration, which he claims is a more important question than that of repair. Among other things, he says:

"Laceration of the perineum should be of very rare occurrence, and a man who finds it a frequent experience in his practice should mend his manner. It is a contradiction of all we know in regard to the processes of nature that she cannot make a perineum that is able to stand the stress and strain of labor. Labor is a physiological process upon which depends the continued existence of the species. It would be the veriest twaddle to assert that the necessary organs were unable to do their legitimate work without injury. The natural forces which, through countless ages, have molded the human body into what it is, have produced the best results which were possible under the circumstances. The female perineum was not constituted by fiat, but was evolved. It came to be what it is through the necessities of the case. It was made for work; through unnumbered thousands of years it has been doing work; and by that work it has been fitted for its work. These be but truisms, yet they are ignored in our text-books and in the lectures of some at least of our professors. Any woman now living is the descendant of a long ancestry of women, all of whom have been mothers. Her organs of generation have been adapted by long process of generation to the purpose of generation. There is no more occasion for her organs being injured in the performance of their natural duties than there is for the male organs of generation to be injured in the performance of their natural duties."

Those of our readers who do obstetrical work will find the *Homœopathic Journal of Obstetrics, Gynaecology, and Pedology* of real value to them.

